# Experimental and Simulation Analysis of Radiation of the Beta Emitting Sources in a Magnetic Field

**DOI:** 10.4274/mirt.30932

**Published:** 2017-06-01

**Authors:** Berrin Çavuşoğlu, Selda Sucu, Hatice Durak, Kadir Akgüngör, Hakan Epik, Türkan Ertay

**Affiliations:** 1 Dokuz Eylül University Health Sciences Institute, Department of Medical Physics, İzmir, Turkey; 2 Dokuz Eylül University Faculty of Medicine, Department of Nuclear Medicine, İzmir, Turkey; 3 Dokuz Eylül University Faculty of Sciences, Department of Physics, İzmir, Turkey

**Keywords:** Beta radiation, Magnetic field, Geant4 Monte Carlo simulation, yttrium-90

## Abstract

**Objective:**

The behavior of beta particles under the magnetic field was investigated both theoretically and experimentally based on the assumption of reducing the damage to the normal tissues created by using magnetic field in radionuclide therapy.

**Methods:**

A water-filled spherical medium and a beta particle source was formed by using Geant4 simulation software for the theoretical study. After applying a homogenous magnetic field, the volume of points at which the particles interact with the medium was calculated by determining particle range. The range of beta particles was examined using yttrium-90 source and Gafchromic films for the experimental study. The setup was kept in normal room conditions and in the magnetic resonance imaging device. Then the irradiated films were analyzed by creating isodose curves.

**Results:**

With the increase of the magnetic field, the number of hits at the center was increased, but the number of hits at the outer boundaries decreased inversely proportional to the strength of the magnetic field. The change perpendicular to the magnetic field was greater as compared to the change parallel to the magnetic field. The volume of hits of beta particles got smaller with the increase of the magnetic field.

**Conclusion:**

When magnetic field is increased, the decrease in the number of interactions at the outer boundaries became more pronounced in the perpendicular direction to the magnetic field. The effect of magnetic field was more apparent for higher energy beta particles than lower energy particles.

## Abstract (tr)

**Amaç:**

Beta yayan radyoizotoplarla yapılan kanser tedavilerinde manyetik alan kullanılarak normal dokularda oluşan zararın azaltılmasının mümkün olabileceği varsayımı ile beta parçacıklarının manyetik alandaki davranışları teorik ve deneysel olarak araştırıldı.

**Yöntem:**

Teorik çalışmada, Geant4 programında içi su dolu küresel bir ortam oluşturuldu ve merkezine beta parçacığı kaynağı yerleştirildi. Homojen bir manyetik alan uygulanarak parçacıkların ortamla etkileştikleri noktaların hacmi, parçacıklarının menzilleri belirlenerek hesaplandı. Deneysel çalışmada, beta parçacıklarının menzili itriyum-90 kaynağı ve Gafkromik film kullanılarak incelendi. Düzenek normal oda koşullarında ve manyetik rezonans görüntüleme cihazında bekletildi. Işınlanan filmler izodoz eğrileri oluşturularak analiz edildi.

**Bulgular:**

Manyetik alan artmasıyla beraber merkezde etkileşim sayısında artma olduğu, merkezin dışında ise etkileşim sayısının manyetik alanla ters orantılı olarak azaldığı görüldü. Manyetik alana dik yöndeki değişim manyetik alana paralel yöndekine göre daha fazla idi. Manyetik alanın artmasıyla beta parçacıklarının etkileştiği hacim küçülmekte idi.

**Sonuç:**

Manyetik alan arttıkça, manyetik alana dik yönlerde merkezin dışındaki alanlarda etkileşim sayısındaki azalım daha belirgin olmaktadır. Manyetik alan etkisinin düşük enerjililere oranla yüksek enerjili beta parçacıklarında daha belirgin olduğu görülmüştür.

## INTRODUCTION

Radionuclide therapy utilizes ionizing radiation for the treatment of undesired tissues such as tumors or an over active thyroid gland. For this purpose, radiation dose is given to the target tissue using radionuclides emitting β-, a+ or Auger electrons. Beta emitters are locally used in prostate seed implants and coronary artery stents, or are systemically administered for the treatment of bone metastases, ablation of thyroid tissue, radioimmunotherapy, etc (1).

Beta particles lose their energy like other charged particles through ionization and cause excitation in soft tissue or water (2). They deviate from their paths as a result of their interaction with atomic nuclei and electrons on their way, depending on the type of medium and the energy of the beta particle (3).

In smaller tumors, the range of beta particles may be greater than the lesion size and only some of their energy accumulates in target cells. Thus, the remaining energy of the ionizing radiation administered may cause damage to normal cells since the surrounding healthy cells cannot be protected and the energy is absorbed by healthy cells as well (1).

The damage to healthy cells causes undesirable side-effects due to the long range of beta particles. The application of a strong magnetic field has been suggested for reducing the particle range in a wide variety of applications of beta radiation. The charged particle entering the magnetic field traces circular paths with the effect of magnetic force (4). Considering the change in the paths of charged particles in the magnetic field, it may be possible to prevent beta particles to leave the target tissue, thus reducing the side effects and increasing the dose to the center, which in turn would increase therapeutic effectiveness. This effect of the magnetic field has been shown in a few studies to enhance the radiation dose absorbed by tumors (5,6) and to protect bone marrow (7).

The theoretical study of the change in the paths of beta particles can be performed by using a computer simulation. In this study, we simulated the course of the interaction of beta particles with matter in magnetic field using Geant4. Geant4 is a Monte Carlo-based particle simulation program developed at CERN, which models and simulates the interaction of different particles within matter (8).

The main goal of this approach was to seek a means of reducing the damage to normal cells during nuclear medicine procedures. For this reason, in this study, the movements of beta particles in magnetic field were investigated with the assumption that it is possible to change both the irradiated volume and the dose by using magnetic field in radionuclide therapy.

## MATERIALS AND METHODS

### Simulation

The geometrical dose distribution of beta (β-) radiation with 0.5-2 MeV energy from a point source in a medium, with different magnetic field strengths (0-3 T) was simulated and analyzed.

Initially, a water-filled spherical medium with a radius of 1.5 cm in which radiation will be detected was created for the simulation. Then a particle source was placed at the center where the beta particles will be thrown mono energetically in random directions. Physical processes and type of interaction of the particles with matter within the medium were determined and included in the simulation. Geometrically homogenous magnetic field was applied in x-direction and then y-axis was chosen to assess the effect of magnetic field on the range of the particles.

To obtain various data including as the positions and energies of the beta particles, slices of 0.1 mm thickness were formed on y-axis so as to be separated 1.0 mm from each other.

Information about the particle at different coordinates where it interacts with the medium was written on text files for each slice. The simulation was done for 106 beta particles with the energies of 0.5, 1, 1.5 and 2 MeV at different magnetic fields, namely 0, 0.5, 1, 1.5, 2, 2.5 and 3 T.

The data including interactions, positions and energies of each slice were recorded from the simulation program Geant4. To analyze the data, each slice was divided into stripes of 0.5 mm apart in both directions, perpendicular (z-axis) and parallel (x-axis) to the magnetic field.

By counting the number of hits in each stripe, we were able to know the change in the amount of interactions starting from the center of the source and also the shape of the interaction volume.

These analyses were done for all the above-mentioned beta energies and magnetic fields.

With these analyses, we measured the dimensions of the volume consisting of the hit points with the medium. The volume was considered to be an ellipsoid and the dimensions were calculated by determining the ranges of particles on each axis. Then the change in the volume of interaction was compared for each energy level and different magnetic fields. This simulation study was approved by the local ethical committee (122/2009).

### Experiment

We used high energy beta (β-) emitter yttrium-90 (^90^Y) glass microspheres to assess the effect of magnetic field on the range of beta particles. ^90^Y decays by beta emission with end-point energy of 2.28 MeV with a mean of 0.93 MeV and a half-life of 64.1 h (9). The maximum range of the ^90^Y beta radiation in water is 11 mm with a mean of 2.5 mm (10).

The range of beta particles was examined using Gafchromic EBT (Beam Therapy) films. Gafchromic EBT radiachromic film dosimeters are used to measure absorbed dose as a function of position in the phantom with a dose range of 2-800 cGy. When exposed to radiation, the color changes by photoionization from colorless to deep blue as a function of absorbed dose (11,12,13).

1.5 T Philips Achieva magnetic resonance imaging (MRI) scanner (Philips Medical Systems, Best, The Netherlands) was used as the source for high magnetic field.

**Figure 1:**
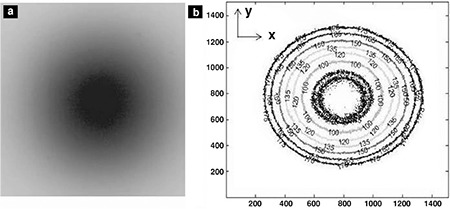
The images of (a) an irradiated film and (b) the isodose curves created by Matlab are shown as an example. The magnetic field is applied on x-axis

The experimental setup was made of plexiglas. 6.4 mCi ^90^Y radioactive source was put into a capillary tube and the lower end of the tube was used as a point source. The radiochromic film was cut into small pieces and put into setup as perpendicular to the capillary tube. Then the setup was placed in water filled container. The first film was exposed for 1 hour, and then the number of decays was calculated as 4.25x10^11^. Each film was kept in normal room conditions and exposed to the same number of decays (4.25x10^11^) without magnetic field (0 T) or in the MRI scanner (1.5 T). The experiment was performed at 0 T and 1.5 T for different distances between the source and the film (0 mm and 2 mm) and repeated four times for each. The irradiated films were analyzed using Matlab Image Processing Toolbox by creating isodose curves (Figure 1). The diameters of isodose curves were measured on each axis, parallel (x-axis) and perpendicular (y-axis) to the magnetic field, to calculate the amount of reduction in irradiated areas at magnetic field. This experimental study was approved by the Dokuz Eylül University Local Ethical Committee (121/2009).

## RESULTS

### Geant4 Simulation

**Figure 2:**
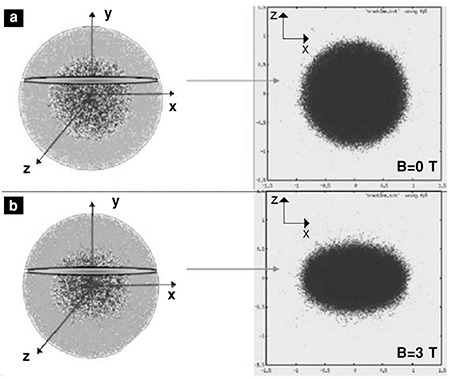
The 3D simulation images of water medium by 106 beta particles with 2 MeV energy from the point source and the images of 2D slices on xz-plane, (a) without magnetic field and (b) with 3 T magnetic field. The shown example slices are taken 2 mm from the center

**Figure 3:**
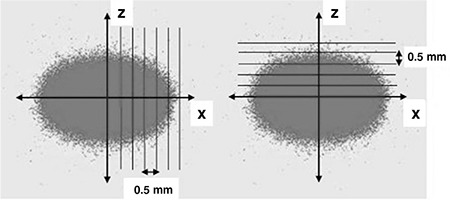
The calculation of hit numbers with 0.5 mm distances along the x-axis (on the left hand side) and the z-axis (on the right hand side) for analyzing the data obtained from Geant4 program****

Simulation revealed that the beta particles stayed closer to the source due to the magnetic force as the magnetic field increased, thus the interaction range was reduced (Figure 2, 3).

**Figure 4:**
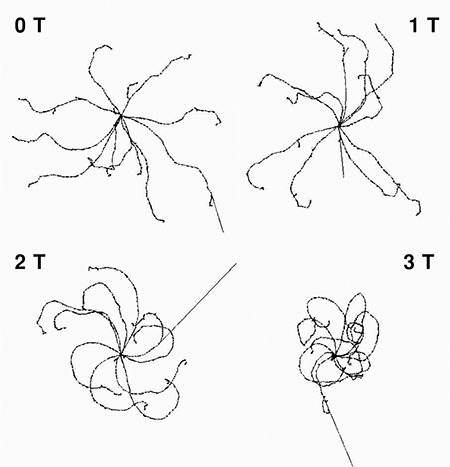
The 3D images of the paths followed by 10 beta particles with 2 MeV of energy from the source in 0 T, 1 T, 2 T, 3 T magnetic fields are shown as an example of the simulation. The magnetic field is applied perpendicular to the page

The paths of a few representative 2 MeV beta particles in different magnetic fields in x-direction are shown in Figure 4. It is seen that the beta particles are localized to the center as the magnetic field increased.

Beta particles changed their directions and moved towards the x-axis under the magnetic field. With the increase of the magnetic field, ranges of the particles were perpendicularly shortened.

**Figure 5:**
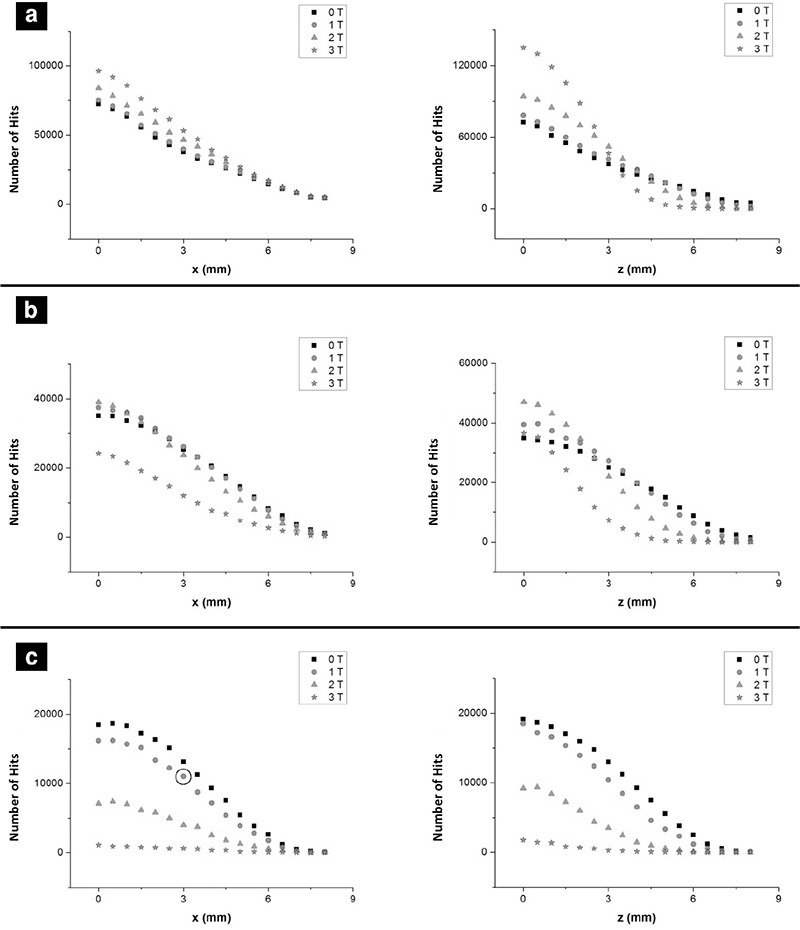
Number of hits of betas with 2 MeV energy under 0 T, 1 T, 2 T and 3 T magnetic fields on the x-axis of the field (on the left) and on the z-axis of the field (on the right) for the slices in (a) 2 mm (b) 4 mm (c) 6 mm from the center. In this figure each point represents the number of hits at a certain stripe of a slice

The hit numbers of beta particles with 0.5, 1, 1.5, 2 MeV energies in different slices were calculated. To represent the results, only 2 MeV energy beta particles at different magnetic fields are shown (Figure 5).

In the inner slice (2 mm) from the center, the number of hits was increased on the x-axis with the amplification of the magnetic field. However, the number of hits was sharply decreased beyond 3 mm on the z-axis. Since the shape of the interaction area was elliptical, the number of hits was increased by 20% for 3 T at the center. On the other hand, the area was the same as the shape of the sphere for 0 T, as expected. Figure 5 (c) also shows that the increased magnetic field reduced the number of hits rapidly. As a result, the counts vanished at higher magnetic fields at the outmost slice.

We calculated the interaction volumes and found that they were reduced by 16% for 1 MeV, 31% for 2 MeV at 2 T relative to zero magnetic field, as seen in Table 1. It should be also noted that the reduction in the volume was much larger at higher magnetic fields. For example, it was reduced by 35% for 1 MeV and 53% for 2 MeV at 3 T. The ratio of the volumes with and without magnetic field is shown in Table 1.

As shown in Table 1, the volume got distinctly smaller as the magnetic field increased, which effect on the volume was with the increase in beta energy.

### Experiment

**Figure 6:**
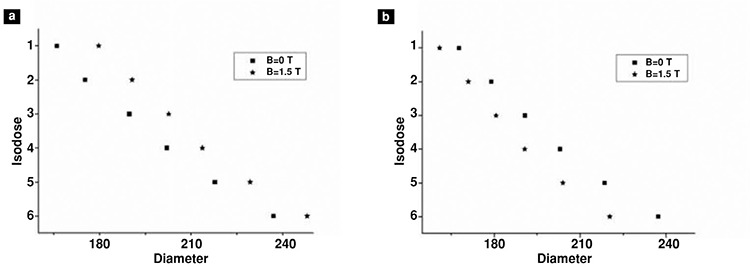
The graph of isodose curves on (a) x-axis and (b) y-axis at 0 T and 1.5 T for 0 mm shows that diameters of isodose curves are increased on x-axis and decreased on y-axis with the effect of the magnetic field

**Figure 7:**
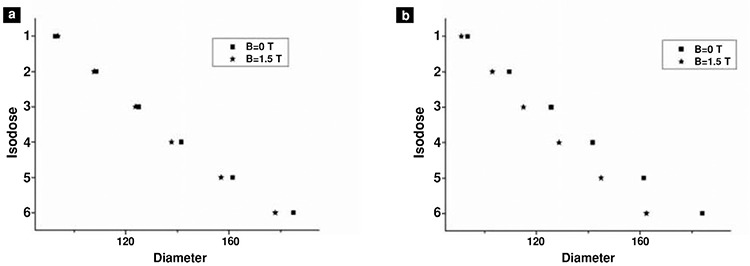
The graph of isodose curves on (a) x-axis and (b) y-axis at 0 T and 1.5 T for 2 mm shows that diameters of isodose curves are decreased especially on y-axis with the effect of the magnetic field

Mean diameters of isodose curves measured on 4 irradiated films were used to assess the effect of magnetic field (Table 2, 3). Mean values of diameters were compared for 0 T and 1.5 T for each isodose curve, from 1 (innermost isodose curve) to 6 (outermost isodose curve) (Figure 6, 7).

When magnetic field was applied, the beta particles were seen to be localized to the center on x-axis (parallel to the magnetic field). The diameter of isodose curves close to the center was increased by 5%. On the other hand, the outer isodose curves were reduced by 8% on y-axis (perpendicular to the magnetic field).

As seen in Figure 7, we couldn’t find any significant difference on x-axis at 2 mm distance from the source when magnetic field was applied. However, diameter of isodose curves of irradiated films was reduced by 13% under magnetic field.

## DISCUSSION

Ionizing radiation is widely used to treat malignant cells. Numerous novel treatments have been introduced by the internal administration of radiopharmaceuticals called targeted radionuclide therapy, in which radiolabeled molecules are specifically targeted to apply high radiation dose to the cancerous cells while striving to give minimum dose to the healthy cells through the use of the features of radiopharmaceutical uptake mechanisms (14). Radionuclide therapy is based on the use of pharmaceuticals as carriers of radionuclides to their target molecule on the surface of tumor cells. Radiation causes irreversible DNA damage and induces cell death through cross-fire irradiation (15). The main objective of the radionuclide therapy is the delivery of radionuclides to tumor cells without any risks for healthy cells (16).

The treatment response of tumors may be insufficient if the targeted lesion dose is lower than required (17). The ranges of beta particles used in these treatments can exceed the size of the target tissue, therefore causing the so called crossfire effect to surrounding cells, which is sometimes undesirable if they are healthy cells. Other problems include the heterogeneity of dose (18) and the dose limiting factors such as bone marrow toxicity (19). Because the path of beta particles changes in the magnetic field, leaving the target tissue can be prevented and it may be possible to reduce the damaging effects to the surrounding healthy tissues, while increasing the radiation effect at the center.

The application of a strong static magnetic field can be used for the benefit of nuclear medical applications. Static homogeneous magnetic field exerts a force on a charged particle and changes the paths of particles. This force, known as the Lorentz force, causes the path of charged particle to curve about the field’s axis thus resulting in a helical path (20). This method has been used to increase the resolution of positron emission tomography (PET) scanners (4,21,22,23,24,25). The positron (β+) is the antiparticle of the β-, and has the same properties as the β- except its electric charge. In magnetic field, the positron moves in circular paths like the β- particle, but in opposite directions due to its opposite charge (26).

In the study carried out by Raaijmakers et al. (27), the effect of magnetic field to the dose was examined experimentally in an MRI-accelerator, and the findings were compared with the results of the Geant4 simulation. It was shown that dose effects of magnetic field can be modeled using Geant4 and that Geant4 is a suitable Monte Carlo code to study the effect of magnetic field on dose distribution for MRI-accelerator.

Wirrwar et al. (21) evaluated the potential effects of magnetic field on shortening the ranges of high energy positrons in PET. Geant simulation model was found suitable and it was reported that 4.5 T homogenous static magnetic field increased the spatial resolution in PET by reducing the high energy positron range. In another study performed by Desbrée et al. (22), the authors examined their previously developed beta microprobe in magnetic field since the combination of nuclear magnetic resonance with PET has become a current issue. The efficiency of the probe for each isotope was investigated by simulating the effect of magnetic field on the ranges of positrons with Geant4. Similarly, in this study, magnetic field shortened the ranges and decreased the volume of interaction of the positrons. In another study carried out by Christensen et al. (23), it was experimentally demonstrated that the spatial resolution in PET images was improved, because positrons were annihilated in a place closer to their point of origin in the strong magnetic field. Similar findings were reported in other simulation studies performed by Iida et al. (24), Rickey et al. (25) and Raylman et al. (4).

Raylman and Wahl (6) showed a reduction in the accumulated dose in normal tissues in radionuclide treatment using different radioisotopes at 10 T with computer simulation. In their other simulation study, they showed a decrease in the deposited bone marrow dose at 10 T during treatment of bone tumors (7). In their experimental cell culture study, they also found a reduction in the number of living lymphoma cells after irradiation at 7 T (28). They claimed that the presence of a strong magnetic field makes treatment of small tumors more effective and decreases the radiation dose to normal tissues.

In this study, we showed that when magnetic field is applied, beta particles deviated from their paths resulting in accumulation of the radiation in the center of the source with a decrease in outer boundaries. In the simulation study, we found that the reduction in the irradiated volume is much larger at higher magnetic fields with 9% at 1 T, 31% at 2 T and 53% at 3 T for 2 MeV. We also showed the effect of magnetic field experimentally. For this purpose, we set an experimental design close to the simulation. However, there were a few differences between methods, such as used sources, shape of sources, tube glass between radiation and water. For this reason, we couldn’t compare the findings obtained from the two methods since absolute particle range was not calculated. However, we were able to observe the geometric distribution of the radiation in two dimensions, parallel and perpendicular to the magnetic field, like in the simulation. We experimentally showed that at the distance of 0 mm from the source, the change parallel to the magnetic field at the inner isodose curves was greater by 5% when magnetic field was applied as compared to the isodose curves of the films irradiated without magnetic field. At 2 mm distance from the source to the direction perpendicular to the magnetic field, there was a reduction of approximately 13% as compared to the change of the diameters of isodose curves of the films irradiated without magnetic field.

The shortcomings of this study include limited number of experiments due to the short half-life of 90Y source. Also, we could obtain only a few data at 0 and 2 mm distance from the source because of the short range of beta particles. In addition, we could not compare the simulation findings with experimental data, as different energy sources were used in the simulation and the experimental study.

## CONCLUSION

In this study, the magnetic field’s effect on beta particles in tissue equivalent water was investigated by simulation of the movements of beta particles in magnetic field using Geant4 program and observed experimentally using gafchromic film irradiated by beta emitter, based on the assumption that the volume of interaction of beta radiation is reduced thus causing more localized damage on the target tissue.

The beta particles tended to have circular movements in the magnetic field and their ranges were shortened when the magnitude of magnetic field was increased.

We found that beta particles tracing outward the point source in random directions were scattering in spherical geometry in the medium, and that the geometrical distribution becomes elliptical when magnetic field is applied because of the shortening of the ranges of beta particles perpendicular to the magnetic field. The shortening in the ranges of particles increases as the magnitude of magnetic field is increased. Therefore, particles from the source accumulate more in the center when magnetic field is applied. This causes the radiation dose to condense in the center. Thus, as the applied magnetic field increases, the irradiated volume gets smaller, the particle hit number per unit length increases close to the center.

In conclusion, radiation may be focused and irradiation of the normal tissues can be prevented while increasing the target dose in the treatments with radioactive isotopes. Although it is not possible to use strong magnetic field in the clinical targeted therapy applications today, it may be possible in the future to shape the distribution of beta particles on the target tissue through magnetic field as desired.

## Figures and Tables

**Table 1:**
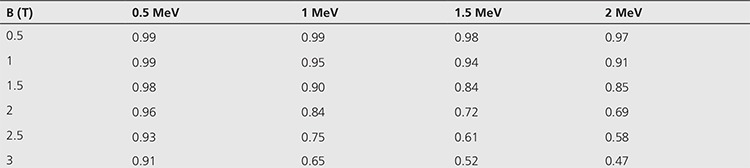
Ratio of volumes for different magnetic fields (0-3 T) for beta particles with 0.5-2 MeV energy

**Table 2:**
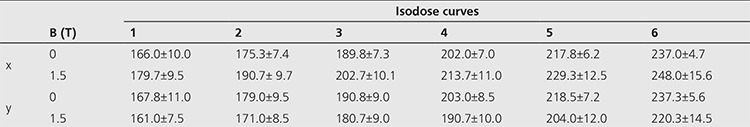
Mean and standard deviation of diameter of isodose curves at 0 and 1.5 T for 0 mm distance from the source on x-axis (parallel to the magnetic field) and y-axis (perpendicular to the magnetic field)

**Table 3:**
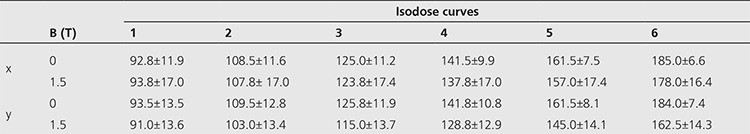
Mean and standard deviation of diameter of isodose curves at 0 and 1.5 T for 2 mm distance from the source on x-axis (parallel to the magnetic field) and y-axis (perpendicular to the magnetic field)
